# Tobacco smoking and methylation of genes related to lung cancer development

**DOI:** 10.18632/oncotarget.10007

**Published:** 2016-06-14

**Authors:** Xu Gao, Yan Zhang, Lutz Philipp Breitling, Hermann Brenner

**Affiliations:** ^1^ Division of Clinical Epidemiology and Aging Research, German Cancer Research Center (DKFZ), D-69120 Heidelberg, Germany; ^2^ Division of Preventive Oncology, German Cancer Research Center (DKFZ) and National Center for Tumor Diseases (NCT), D-69120 Heidelberg, Germany; ^3^ German Cancer Consortium (DKTK), German Cancer Research Center (DKFZ), D-69120 Heidelberg, Germany; ^4^ Pneumology and Respiratory Critical Care Medicine, Thoraxklinik, University of Heidelberg, D-69126 Heidelberg, Germany

**Keywords:** DNA methylation, tobacco smoking, lung cancer, whole blood sample

## Abstract

Lung cancer is a leading cause of cancer-related mortality worldwide, and cigarette smoking is the major environmental hazard for its development. This study intended to examine whether smoking could alter methylation of genes at lung cancer risk loci identified by genome-wide association studies (GWASs). By systematic literature review, we selected 75 genomic candidate regions based on 120 single-nucleotide polymorphisms (SNPs). DNA methylation levels of 2854 corresponding cytosine-phosphate-guanine (CpG) candidates in whole blood samples were measured by the Illumina Infinium Human Methylation450 Beadchip array in two independent subsamples of the ESTHER study. After correction for multiple testing, we successfully confirmed associations with smoking for one previously identified CpG site within the *KLF6* gene and identified 12 novel sites located in 7 genes: *STK32A*, *TERT*, *MSH5*, *ACTA2*, *GATA3*, *VTI1A* and *CHRNA5* (FDR <0.05). Current smoking was linked to a 0.74% to 2.4% decrease of DNA methylation compared to never smoking in 11 loci, and all but one showed significant associations (FDR <0.05) with life-time cumulative smoking (pack-years). In conclusion, our study demonstrates the impact of tobacco smoking on DNA methylation of lung cancer related genes, which may indicate that lung cancer susceptibility genes might be regulated by methylation changes in response to smoking. Nevertheless, this mechanism warrants further exploration in future epigenetic and biomarker studies.

## INTRODUCTION

Lung cancer is the most common cancer and a leading cause of cancer-related mortality globally [1]. In recent years, several large genome-wide association studies (GWASs) have been conducted to identify genetic risk factors of lung cancer [2]. They have successfully identified numerous single-nucleotide polymorphisms (SNPs) that might play a role in the pathophysiology of lung cancer, such as loci located in chromosomal regions 15q (nicotinic acetylcholine receptor subunits: *CHRNA3*, *CHRNA5*), 5p (*TERT-CLPTM1L*) and 6p (*BAT3-MSH5*).

Smoking, the best established environmental hazard of lung cancer, accounts for 80% of the worldwide lung cancer burden in males and at least 50% in females [1]. Recent studies have shown that smoking could interact with genetic variation to influence lung cancer, including lung tumor initiation and progression [3, 4]. DNA methylation, which could be employed as a useful and stable surrogate of the genetic response, has recently been suggested to be one of the potential mechanisms of such interaction for smoking-related health outcomes [5, 6].

Recently, a number of epigenome-wide association studies (EWASs) have established the important role of tobacco smoking in genomic DNA methylation profiles within whole blood samples. They identified smoking related CpG sites in various genes, such as *AHRR*, *F2RL3* and *GPR15*, in whole blood samples, and showed that these sites could be utilized as quantitive biomarkers of current and past smoking exposure and predictors of smoking-associated health risks [5–8]. Another two studies by Steenaard et al. and Ligthart et al. have demonstrated that smoking is associated with differential DNA methylation of the risk genes of coronary artery disease and diabetes [9, 10]. However, no previous studies have systematically addressed the impact of smoking on DNA methylation of risk loci for lung cancer. Hence, we conducted an epigenetic investigation in the ESTHER study, focusing on the association of smoking with whole blood DNA methylation of loci at/near confirmed lung cancer related genes, with the aim of identifying methylation signals that could have the potential to aid in the development of risk prediction models or in advancing the understanding of the exact links of smoking with lung cancer.

## RESULTS

### Participant characteristics

Characteristics of the study population in the discovery (n=978) and validation panels (n=531) were comparable with respect to age, lifestyle factors, smoking behavior, as well as prevalent diseases, and are summarized in Table 1. Average age in the two subsets was about 62 years. More than half of the participants in each subset were ever smokers, and around 18% still smoked at the time of recruitment. In both subsets, the proportions of men were much higher in current smokers than that in never smokers: 60.8% vs. 29.4% in the discovery panel and 48.0% vs. 21.1% in the validation panel (data not shown). Average cumulative smoking exposure in current smokers and former smokers were 36.8 and 23.3 pack-years, respectively, in the discovery panel, and 33.9 and 19.9 pack-years, respectively, in the validation panel. Average cessation time for former smokers in the two subsets was also similar, approximately 17 years.

**Table 1:** Characteristics of study population in discovery and validation panels ^a^

Characteristics	Discovery Panel	Validation Panel	*p* value
**N**	978	531	
**Age (years)**	62.1 (6.5)	62.0 (6.6)	0.817
**Sex**			<0.001
Male	495 (50.6%)	207 (39.0%)	
Female	483 (49.4%)	324 (61.0%)	
**Smoking status**			0.877
Current smoker	181 (18.5%)	98 (18.4%)	
Former smoker	328 (33.5%)	182 (34.3%)	
Never smoker	469 (48.0%)	251 (47.3%)	
**Body mass index ^b^**			0.246
Underweight (<18.5)	8 (0.8%)	1 (0.2%)	
Normal (18.5-<25.0)	237 (24.3%)	161 (30.3%)	
Overweight (25.0-<30.0)	472 (48.4%)	228 (42.9%)	
Obese (≥30.0)	258 (26.5%)	141 (26.6%)	
**Alcohol consumption ^c^**			0.511
Abstainer	311 (34.1%)	169 (34.4%)	
Low	531 (58.2%)	290 (59.1%)	
Intermediate	53 (5.8%)	27 (5.5%)	
High	17 (1.9%)	5 (1.0%)	
**Physical activity ^d^**			0.061
Inactive	189 (19.3%)	109 (20.5%)	
Low	433 (44.3%)	261 (49.2%)	
Medium or high	356 (36.4%)	161 (30.3%)	
**Prevalence of diabetes ^e^**			0.647
Not prevalent	819 (84.4%)	436 (83.5%)	
Prevalent	151 (15.6%)	86 (16.5%)	
**Prevalence of CVD ^f^**			0.627
Not prevalent	796 (81.5%)	438 (82.5%)	
Prevalent	181 (18.5%)	93 (17.5%)	
**Prevalence of cancer ^g^**			0.748
Not prevalent	892 (93.4%)	487 (93.8%)	
Prevalent	63 (6.6%)	32 (6.2%)	
**Leukocyte composition^h^**			
CD8+ T-cells	0.081 (0.039)	0.098 (0.041)	<0.001
CD4+ T-cells	0.166 (0.058)	0.171 (0.056)	0.041
NK cells	0.098 (0.044)	0.096 (0.042)	0.281
B-cells	0.063 (0.024)	0.070 (0.019)	<0.001
Monocytes	0.101 (0.022)	0.100 (0.020)	0.867
Granulocytes	0.548 (0.097)	0.531 (0.094)	0.002
**Pack-years of smoking^i^**			
Current smokers	36.8 (19.3)	33.9 (17.5)	0.250
Former smokers	23.3 (16.3)	19.9 (15.1)	0.031
**Smoking cessation time (years) ^j^**	17.3 (11.3)	17.6 (10.6)	0.755

a Mean values (SD) for continuous variables and n (%) for categorical variables; Kruskal-Wallis Test was applied to examine continuous variables and Chi-Square test was applied to examine categorical variables

b Data missing for 3 participants in discovery panel

c Data missing for 66 and 40 participants, respectively, in discovery and validation panels. Categories defined as follows: abstainer, low [women: 0 −<20 g/d, men: 0 −<40 g/d], intermediate [20 −<40 g/d and 40 −<60 g/d, respectively], high [≥40 g/d and ≥60 g/d, respectively]

d Categories defined as follows: inactive [< 1h of physical activity/week], medium or high [≥2 h of vigorous and ≥ 2 h of light physical activity/week], low [other]

e Data missing for 8 and 9 participants, respectively, in discovery and validation panels

f CVD: cardiovascular disease. Data missing for 1 participant in discovery panel

g Data missing for 23 and 12 participants, respectively, in discovery and validation panels

h Estimated by the Houseman algorithm [27]

i A pack-year was defined as having smoked 20 cigarettes per day for 1 year, including all participants from validation panel, pack-year= 0 for never smokers

j Former smokers only, data missing for 9 and 3 participants, respectively, in discovery and validation panels; cessation time equals age at recruitment minus age at cessation

### Associations between tobacco smoking and methylation of lung cancer related genes

**Figure 1:**
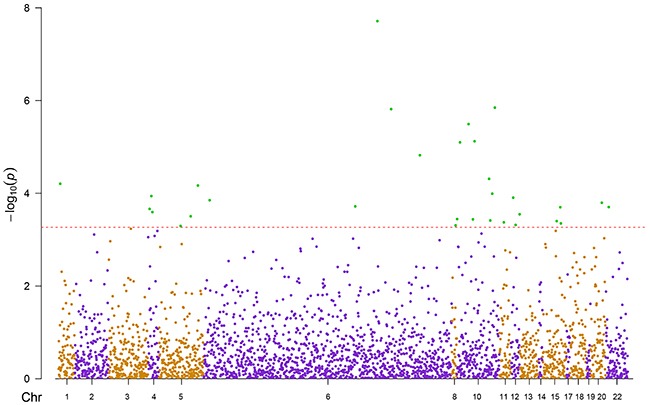
Manhattan plot of discovery panel Red line: raw p-value of FDR = 0.05; Green dots: 31 significant sites; Chr: chromosome position

DNA methylation levels of 2854 CpG candidates corresponding to 75 genes were measured by the Illumina Infinium Human Methylation450 Beadchip array. Associations between current smoking exposure (current vs. never; independent variable) and methylation levels of these candidates (dependent variable) were assessed by three mixed linear regression models (Models 1- 3) with methylation assay batch as random effect and increasing adjustment for potential confounders (details were presented in Methods). Compared with Model 1 and Model 2 which were less powerful (Supplementary Table S1), after fully controlling for confounding factors (Model 3), 31 of the 2854 CpG candidates passed the threshold of FDR <0.05 in the discovery phase (Figure 1, Supplementary Table S2). The 31 CpG sites were then replicated in the validation panel by the fully-adjusted mixed linear regression model (Model 3). As a result, 13 of these 31 CpG sites were confirmed as significantly smoking-related loci (Table 2, FDR < 0.05). Among these, only cg24287110 (*KLF6*), was previously reported to be related to smoking exposure [11]. The remaining 12 sites were located in 7 genes: *STK32A* (n=1), *TERT* (n=2), *MSH5* (n=2), *ACTA2* (n=1), *GATA3* (n=3), *VTI1A* (n=2) and *CHRNA5* (n=1). Current smoking was mostly associated with hypomethylation (11 sites), whereas hypermethylation was observed at cg17928584 (*STK32A*) and cg19696491 (*CHRNA5*). Effect sizes of the 13 CpG sites between never and current smokers ranged from 0.6% to 2.9%.

**Table 2:** Significant associations between tobacco smoking and methylation of lung cancer related genes in validation panel

CpG site	Gene	Mean β value (Standard deviation)		Effect size ^b^	Estimate (se)	*p*-value	FDR
		Never smoker	Current smoker				
cg00640087	*MSH5*	0.165 (0.036)	0.159 (0.035)	−0.006	−7.4 e-3 (3.1 e-3)	0.019	0.049
cg03281572	*VTI1A*	0.812 (0.028)	0.793 (0.036)	−0.019	−0.018 (3.0 e-3)	3.8 e-7	1.2 e-5
cg07269053	*VTI1A*	0.733 (0.039)	0.715 (0.052)	−0.018	−0.013 (5.0 e-3)	0.007	0.023
cg10163955	*GATA3*	0.669 (0.043)	0.640 (0.049)	−0.029	−0.024 (5.1 e-3)	5.1 e-4	6.6 e-5
cg11430077	*GATA3*	0.147 (0.032)	0.132 (0.029)	−0.015	−0.013 (4.0 e-3)	0.001	0.006
cg12324353	*TERT*	0.788 (0.032)	0.779 (0.032)	−0.009	−0.011 (3.5 e-3)	0.002	0.011
cg17928584	*STK32A*	0.156 (0.053)	0.161 (0.052)	0.005	0.012 (5.0 e-3)	0.020	0.049
cg19335412	*ACTA2*	0.461 (0.036)	0.451 (0.033)	−0.010	−0.011 (4.1 e-3)	0.009	0.026
cg19696491	*CHRNA5*	0.470 (0.058)	0.488 (0.060)	0.018	0.018 (6.9 e-3)	0.010	0.028
cg20640261	*MSH5*	0.443 (0.048)	0.424 (0.048)	−0.019	−0.015 (5.0 e-3)	0.003	0.013
cg22770911	*GATA3*	0.481 (0.033)	0.458 (0.042)	−0.023	−0.015 (4.5 e-3)	0.001	0.005
cg24287110	*KLF6*	0.365 (0.056)	0.349 (0.053)	−0.016	−0.022 (6.0 e-3)	6.2 e-4	0.005
cg24908166	*TERT*	0.926 (0.021)	0.916 (0.026)	−0.010	−0.010 (2.6 e-3)	0.0001	0.001

a Adjusted for age (years), sex, random batch effects, leukocyte distribution (Houseman algorithm [27]), alcohol consumption (abstainer/ low/ intermediate/ high), body mass index (BMI, underweight/ normal weight/ overweight/ obese), physical activity (inactive/ low/ medium or high), prevalence of cardiovascular diseases (yes/no), prevalence of diabetes (yes/no) and prevalence of cancer (yes/no) All 31 loci identified by discovery panel were validated by the three models, and the threshold of FDR is 0.05. A total of 13 CpG sites were validated as significant smoking-related CpG sites by validation

b Effect size = Mean β_current smoker_ – Mean β_never smoker_

Furthermore, in the analyses of associations between other smoking indicators and the 13 validated CpG sites which were identified as the smoking-related loci, all loci except cg19696491 (*CHRNA5*) were significantly associated with pack-years (Table 3, FDR<0.05), whereas none of the 13 loci exhibited an association with the time since smoking cessation after FDR correction. In line with this, comparisons of methylation between current and former, or between former and never smokers generally were weaker, and did not reach significance, with the possible exception of cg19335412 (*ACTA2*) (adjusted *p-*value = 0.018 for the comparison of former and never smokers). However, methylation changes associated with former smoking were generally in the same direction as those associated with current smoking (detailed data not shown).

**Table 3:** Associations of cumulative smoking exposure (pack-years) and cessation time (year) with methylation of validated CpG sites^a^

CpG site	Gene	Cumulative smoking exposure^b^			Smoking cessation time ^c^		
		Estimate (se)	*p*-value	FDR	Estimate (se)	*p*-value	FDR
cg00640087	*MSH5*	−2.3 e-4 (7.2 e-5)	1.4 e-3	1.7 e-3	2.7 e-4 (1.9 e-4)	0.155	0.252
cg03281572	*VTI1A*	−3.8 e-4 (8.8 e-5)	1.6 e-5	5.1 e-5	4.9 e-4 (2.6 e-4)	0.060	0.131
cg07269053	*VTI1A*	−2.5 e-4 (1.0 e-4)	0.015	0.016	2.3 e-4 (3.0 e-4)	0.455	0.493
cg10163955	*GATA3*	−5.5 e-4 (1.1 e-4)	5.8 e-7	3.7 e-6	7.0 e-4 (3.2 e-4)	0.030	0.131
cg11430077	*GATA3*	−3.0 e-4 (8.8 e-5)	8.0 e-4	1.1 e-3	5.3 e-4 (2.4 e-4)	0.032	0.131
cg12324353	*TERT*	−3.1 e-4 (7.3 e-5)	2.3 e-5	6.1 e-5	3.7 e-4 (1.9 e-4)	0.052	0.131
cg17928584	*STK32A*	3.7 e-4 (1.1 e-4)	7.0 e-4	1.1 e-3	−3.5 e-4 (2.9 e-4)	0.230	0.307
cg19335412	*ACTA2*	−3.2 e-4 (9.2 e-5)	6.0 e-4	1.1 e-3	4.4 e-4 (2.5 e-4)	0.084	0.156
cg19696491	*CHRNA5*	2.0 e-4 (1.5 e-4)	0.178	0.178	4.7 e-4 (4.2 e-4)	0.262	0.310
cg20640261	*MSH5*	−5.3 e-4 (1.1 e-4)	2.5 e-6	1.1 e-5	6.4 e-4 (3.1 e-4)	0.040	0.131
cg22770911	*GATA3*	−4.8 e-4 (9.1 e-5)	2.2 e-7	2.9 e-6	6.0 e-4 (2.7 e-4)	0.027	0.131
cg24287110	*KLF6*	−5.7 e-4 (1.4 e-4)	5.1 e-5	1.1 e-4	4.5 e-4 (3.8 e-4)	0.236	0.307
cg24908166	*TERT*	−1.8 e-4 (5.5 e-5)	1.5 e-3	1.7 e-3	4.3 e-5 (1.6 e-4)	0.788	0.788

a Estimated by mixed linear regression in validation panels. Both models were adjusted for age (years), sex, batch effects, leukocyte distribution (Houseman algorithm [27]), alcohol consumption (abstainer/ low/ intermediate/ high), body mass index (BMI, underweight/ normal weight/ overweight/ obese), physical activity (inactive/ low/ medium/ high), prevalence of cardiovascular diseases (yes/no), prevalence of diabetes (yes/no) and prevalence of cancer (yes/no); The threshold of FDR (false discovery rate) is 0.05

b A pack-year was defined as having smoked 20 cigarettes per day for 1 year, including all participants from validation panel, pack-year= 0 for never smokers

c Cessation time defined as age at the time of recruitment minus age at cessation, including former and current smokers from validation panel, cessation time = 0 for current smokers

### Characteristics of significant CpG sites

Genome characteristics of the 13 validated CpG sites are presented in Table 4. They are located at chromosomes 5 (n=3), 6 (n=2), 10 (n=7) and 15 (n=1). Eight of these 13 CpG sites are located at the gene bodies, 4 at the transcription start sites (TSS200/ TSS1500) and only one at the untranslated region (3′UTR). None of them is located at the cis-eQTLs. With the exception of three CpG sites within *GATA3*, the distances between other significant CpG sites and their corresponding lung cancer related SNPs were less than 1Mb. Correlations between methylation at the 13 sites are described in Supplementary Table S3, significant moderate pairwise correlations were frequently observed, stronger positive correlations were seen between CpG sites located on the same genes. In particular, cg19696491 within *CHRNA5* has the strongest correlations (*p*<0.0001) with other CpG sites except loci cg11430077 (*GATA3*) and cg24287110 (*KLF6*).

**Table 4:** Characteristics of the validated CpG sites

CpG site	Position ^a^	Gene	Function	Placement	Reported SNPs	SNP position
cg17928584	chr5:146,614,458	*STK32A*	Encoding members of the serine/threonine kinase family that has a paramount role in cellular homeostasis, transcription factor phosphorylation and cell-cycle regulation	TSS200	rs2895680	chr5:146,643,865-146,644,365
cg12324353 cg24908166	chr5:1,269,197 chr5:1,268,801	*TERT*	Encoding human telomerase reverse transcriptase, which is important in the maintenance of telomere length	BodyBody	rs2736100 rs2853677 rs465498	chr5:1,286,266-1,286,766 chr5:1,286,944-1,287,444 chr5:1,325,553-1,326,053
cg00640087 cg20640261	chr6:31,707,203 chr6:31,707,020	*MSH5*	Encoding a member of the mutS family of proteins that are involved in DNA mismatch repair and meiotic recombination	TSS1500 TSS1500	rs3117582	chr6:31,620,270-31,620,770
cg19335412	chr10:90,694,875	*ACTA2*	Encoding a protein which belongs to the actin family of proteins and are highly conserved proteins that play a role in cell motility, structure and integrity	3′UTR	rs1926203	chr10:90,727,084-90,727,584
cg10163955cg11430077cg22770911	chr10:8,101,402chr10:8,099,019chr10:8,101,307	*GATA3*	Encoding a protein which belongs to the GATA family of transcription factors	BodyBodyBody	rs1663689 ^b^	chr10:9,024,945-9,025,445
cg24287110	chr10:3,824,688	*KLF6*	Encoding a member of the Kruppel-like family of transcription factors, which is a transcriptional activator and functions as a tumor suppressor	Body	rs10508266 rs3750861	chr10:3,839,764-3,840,264 chr10:3,824,183-3,824,683
cg03281572 cg07269053	chr10:114,502,318 chr10:114,497,612	*VTI1A*	Encoding vesicle transport through interaction with t-SNAREs homolog 1A	Body Body	rs7086803	chr10:114,498,226-114,498,726
cg19696491	chr15:78,857,125	*CHRNA5*	Encoding a nicotinic acetylcholine receptor subunit, which is a member of a superfamily of ligand-gated ion channels that mediate fast signal transmission at synapses	TSS1500	rs1051730 ^c^ rs16969968 rs8034191 ^c^	chr15:78,894,089-78,894,589 chr15:78,882,675-78,883,175 chr15:78,805,773-78,806,273

a According to GRCh37/hg19

b This SNP is located close to *GATA3*

c *CHRNA5 is* cis-eQTL gene of this SNP

## DISCUSSION

In the present study, based on two independent subgroups of a population-based cohort of older adults from Germany, we identified 13 smoking-related CpG sites within 8 genes suggested to be associated with lung cancer development by GWASs. Smoking-induced hypomethylation was observed for loci within *KLF6*, *TERT*, *MSH5*, *ACTA2*, *GATA3* and *VTI1A*, and hypermethylation was observed for loci within *STK32A* and *CHRNA5*. The effect sizes between never and current smokers ranged from 0.6% to 2.9%. These findings may indicate that lung cancer susceptibility genes might be regulated by methylation changes in response to smoking. The associations with smoking may also partly explain the positive correlation of methylation levels between the identified sites.

Altogether, we were able to identify 12 novel smoking-related CpG sites and replicate one previously identified locus within two independent cohorts. Although their methylation alterations were not as pronounced as well-established smoking-related CpG sites, such as cg05575921 (*AHRR*) and cg03636183 (*F2RL3*) [8, 12–14], clear patterns of lowest (highest) and intermediate methylation levels, respectively, among current and former smokers, compared with never smokers were consistently observed for all hypomethylated (hypermethylated) loci. Although differences between former and never smokers were weaker and not statistically significant, they were in the same direction as differences between current and never smokers, and additional associations were observed between cumulative smoking exposure and methylation at the identified sites. This pattern of “methylation recovery” after quitting smoking is consistent with findings from recent epigenetic studies of smoking cessation [11, 14, 15]. Accordingly, it appears worthwhile to further explore dose-response relationships of life-time smoking exposure with methylation at the identified loci in larger cohorts.

Our study also discloses evidence that might narrow the apparent ethnical discrepancy of lung cancer susceptibility. We identified methylation changes in three genes, *VTI1A*, *STK32A* and *GATA3* that were rarely reported in relation to lung cancer among Caucasians previously. The corresponding SNP rs7086803 of *VTI1A* (vesicle transport through interaction with t-SNAREs 1A) was only identified in female non-smoking Asians as the strongest association signal of lung cancer [16]. A recent study further identified it as a potential contributor to lung cancer susceptibility and poor survival in smoking Chinese [17], but this locus never demonstrated a significant association with lung cancer in GWASs among other ethnicities. Likewise, *STK32A* (encoding serine/threonine kinase 32A) was only reported by a GWAS in a Chinese population, and the risk allele, rs2895680, was significantly associated with smoking dose [18]. Lastly, for *GATA3* (GATA binding protein 3), no corresponding SNP was disclosed by any GWASs on lung cancer yet, while only an adjacent SNP, rs1663689, was identified in a Chinese population and might mediate genetic damage among workers exposed to polycyclic aromatic hydrocarbons [18, 19]. Overall, our study might provide some indications that these loci may play some roles in the pathway between smoking and lung cancer development in the Caucasian population as well, which should be followed up in further research.

Furthermore, we also identified CpG sites within two well-established lung cancer related genes. *CHRNA5* is one of the three cholinergic nicotine-receptor genes within genome region 15q25, encoding nicotine acetylcholine receptors (nAChRs) in neuronal and other tissues [20]. Its association with smoking quantity was reported in 2008, suggesting that SNPs in nAChRs may alter the risk of lung cancer through smoking behavior and regulate direct effects of nicotine as well [20]. Our finding of hypermethylation of cg19696491 within *CHRNA5* under smoking exposure possibly reflects altered expression of *CHRNA5*, which could render a potential mechanism to support this suggestion. *TERT* (telomerase reverse transcriptase) is another plausible lung-cancer gene candidate which is known for its function in telomere replication and maintenance [21]. It is located at the *5p15.33* region, which is not only involved in lung cancer, but also in brain, bladder and prostate cancer development [22]. Moreover, locus cg12324353 within *TERT* was recently reported to be related to coronary artery disease [9]. These findings indicate that the genotypes and epigenotypes of *TERT* might provide valuable contributions to signatures for risk of a wide range of cancers and chronic diseases, which warrants further exploration. The same applies to another three genes *KLF6* (Krüppel-like zinc finger transcription factor) [23], *MSH5* (MutS protein homolog 5) [24] and *ACTA2* (Alpha-smooth muscle actin) [25], which were also found to be associated with lung cancer by several previous GWASs, albeit not as prominently as *CHRNA5* and *TERT*.

Major strengths of the present study include the relatively large sample size with detailed information on a broad range of covariates in a large population-based cohort and the comprehensive validation in an independent group. Although smoking and lung cancer related changes of methylation would be expected to primarily manifest in buccal tissues [26], we were able to disclose such changes in DNA of whole blood samples, which would be the primary sample matrix available in screening settings in general practice. Even though associations of smoking with DNA methylation in whole blood may be affected by smoking related shifts in leukocyte distribution, the observed associations persisted after control for leukocyte distribution by the Houseman algorithm [27]. Furthermore, even potential (residual) confounding by leukocyte distribution would not impair the potential utility of the methylation patterns for risk prediction. Lastly, one plausible explanation for our observation could be that DNA methylation lies on the regulatory pathway linking smoking with lung cancer, which would be in line with Zhang et al.'s finding that the association between smoking and lung cancer was strongly attenuated or even disappeared when DNA methylation was included in predictive models [28]. Therefore, further studies focusing on elucidating potential causal pathways would be desirable. Still, other alternative/ additional explanations, such as DNA methylation being a more reliable marker of smoking exposure or DNA methylation reflecting susceptibility to smoking exposure would also have to be kept in mind. In addition, genomic variations might influence the DNA methylation patterns identified in our study. However, due to the lack of gene expression data and the limited number of lung cancer cases in our study population, we were not able to address potential underlying pathophysiological mechanisms.

Even with significant strides in diagnosis and treatment, the prognosis of lung cancer remains poor, with overall 5-year survival rates around 15%, primarily owing to detection at advanced stages [29]. Screening by available routine assays like sputum cytological examination and chest radiography, but also by low-dose computed tomography have serious limitations [30, 31]. Therefore, novel approaches for enhanced risk stratification and performance of lung cancer screening would be highly desirable. DNA methylation signatures might be a promising approach toward this end. Recently, Zhang et al. demonstrated the potential of methylation of *F2RL3*, a strongly smoking associated locus, as a predictor of lung cancer risk [28]. Further studies should evaluate the extent to which the identified CpG sites may be more predictive of lung cancer than self-reported smoking indicators or genetic background, and then address the potential of such CpG sites, alone or in combination with other markers, to predict lung cancer risk and to enhance risk stratification and screening for lung cancer.

## MATERIALS AND METHODS

### Study population

All study subjects were selected from the ESTHER study, an ongoing statewide population-based cohort study conducted in southwest Germany. Details of study design have been reported previously [32]. Briefly, 9949 older adults (aged 50-75 years) were enrolled by their general practitioners during a routine health check-up between July 2000 and December 2002, and followed up thereafter. Two independent subgroups were selected as discovery panel and validation panel, respectively, for epigenetic analyses. The discovery panel included 1000 participants who were recruited consecutively at the start of ESTHER study between July and October 2000. The validation panel included 548 participants randomly selected from participants recruited between October 2000 and March 2001. The study was approved by the ethics committees of the University of Heidelberg and the state medical board of Saarland, Germany. Written informed consent was issued by all participants.

### Data collection

Information on socio-demographic characteristics, lifestyle factors, health status, and history of major diseases at baseline was obtained by standardized self-administrated questionnaires. Participants were asked about past and present cigarette, cigar and pipe smoking behavior and were then categorized into current, former and never smokers. Furthermore, detailed information on smoking history was also obtained from questionnaires, including age at initiation and smoking intensities at various ages, as well as age of quitting smoking for former smokers. Twenty-two and seventeen participants were excluded from the discovery and the validation panel, respectively, because of missing information on smoking status, respectively. Additional information on body mass index (BMI) and prevalent diseases, such as diabetes, cancer, or cardiovascular disease was extracted from a standardized form filled by the general practitioners during the health check-ups. Prevalent cardiovascular disease at baseline was defined by either physician-reported coronary heart disease or a self-reported history of myocardial infarction, stroke, pulmonary embolism or revascularization of the coronary arteries. Prevalent cancer [ICD-10 C00-C99 except non-melanoma skin cancer (C44)] was defined by either self-report or records from the Saarland Cancer Registry. Blood samples were taken during the health check-up and stored at −80°C until further processing. Whole blood DNA was extracted by using a salting out procedure [33].

### DNA methylation data

DNA methylation of whole blood samples was assessed by the Illumina Infinium Human Methylation 450 Beadchip array (Illumina, San Diego, CA, USA). As previously described [34], samples were analyzed following the manufacturer's instruction at the Genomics and Proteomics Core Facility of German Cancer Research Center, Heidelberg, Germany. Illumina's GenomeStudio® (version 2011.1; Illumina.Inc.) was employed to extract DNA methylation signals from the scanned arrays (Module version 1.9.0; Illumina.Inc.). Methylation status of a specific CpG site was quantified as a β value ranging between 0 (no methylation) and 1 (full methylation). According to the manufacturer's protocol, no background correction was done and data were normalized to internal controls provided by the manufacturer. All controls were checked for inconsistencies in each measured plate. Signals of probes with a detection *p*-value >0.05 were excluded from analysis. We used the Illumina normalization and preprocessing method implemented in Illumina's Genomestudio (“Illumina normalization”).

### Identification of CpG candidates

**Figure 2:**
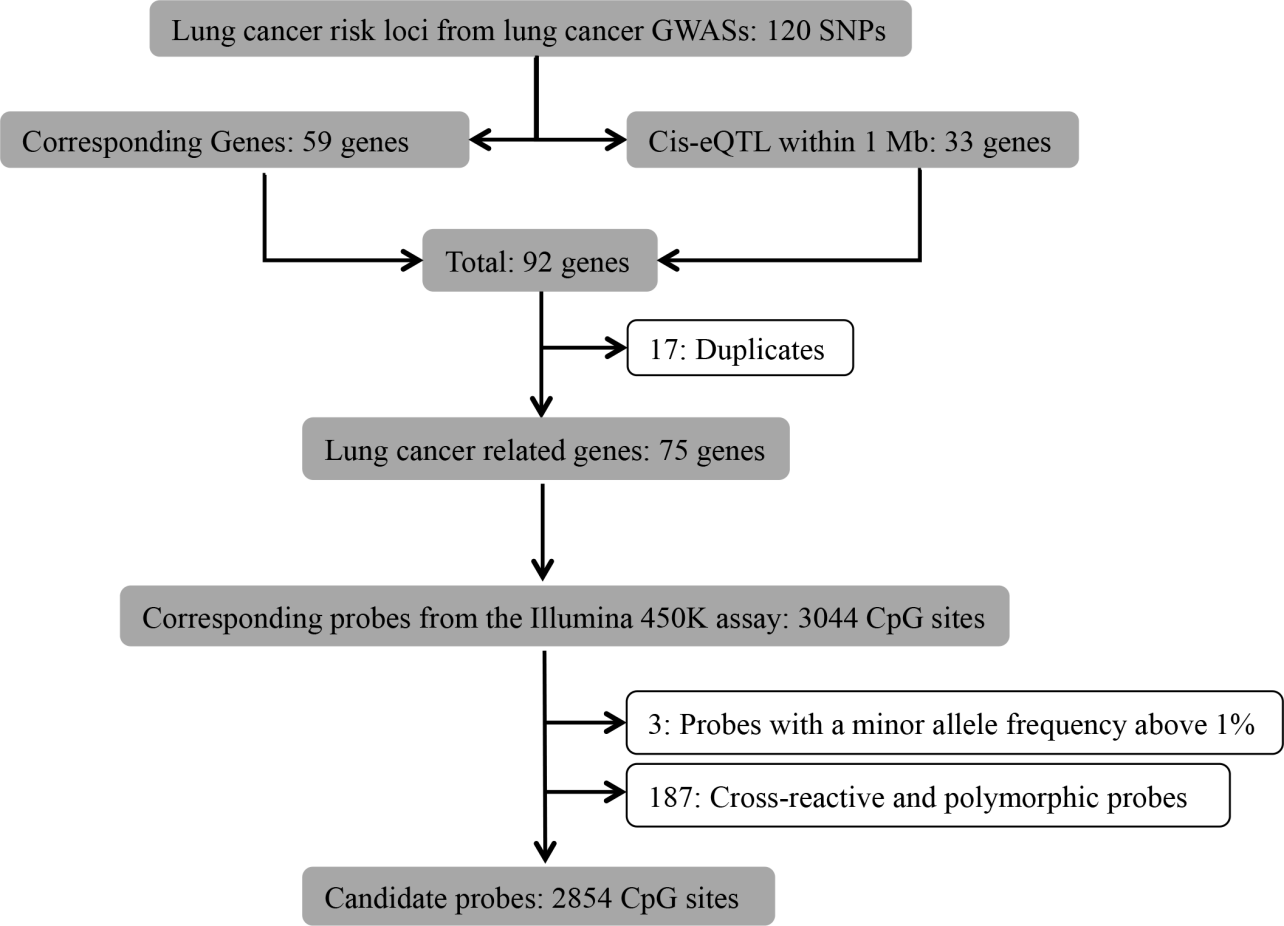
Flowchart of selection of CpG sites

GWASs for lung cancer conducted among smokers, non-smokers and the general population that were published from 2007 to July.2015 [2, 16–21, 23–25, 35–39] were reviewed by one of the authors (XG), from which 120 lung cancer related SNPs within 59 genetic regions were identified (Figure 2). Furthermore, since cis-expression-quantitive trait loci (cis-eQTL) might affect the gene expression levels of nearby genes [40], we therefore identified 33 cis-eQTL within 1 Mb of the identified SNPs from the blood cis-eQTL database (FDR < 0.05) [40]. After excluding 17 duplicates, we identified 3044 corresponding methylation probes within the remaining 75 lung cancer related genes from the probe database of the Illumina 450K assay. Subsequently, we excluded 3 probes containing SNPs with a minor allele frequency above 1% from the candidate list, since variations in these SNPs are able to cause bias in the methylation measurement [41]. We also excluded known cross-reactive and polymorphic probes (n=187), as they could introduce bias in the results [42]. Finally, we obtained a list of 2854 probes considered for further analysis (Supplementary Table S1).

### Statistical analysis

The study populations in the discovery and validation panels were described with respect to major socio-demographic characteristics, lifestyle factors, smoking behavior and prevalent diseases.

Firstly, we chose the current and never smokers from the discovery panel to investigate the associations between current smoking exposure (current vs. never; independent variable) and methylation levels of 2854 CpG candidates (dependent variable). Three mixed linear regression models with methylation assay batch as random effect were employed, controlling for potential confounding factors, including factors that have been shown to be associated with DNA methylation in previous studies [43–47]. Model 1 was adjusted for age (years) and sex. Model 2 was additionally adjusted for the leukocyte distribution estimated by the Houseman algorithm [27]. Model 3 was further adjusted for alcohol consumption (abstainer, low [women: 0 −<20 g/d, men: 0 −<40 g/d], intermediate [20 −<40 g/d and 40 −<60 g/d, respectively], high [≥40 g/d and ≥60 g/d, respectively]), body mass index (BMI, kg/m^2^, underweight [<18.5], normal weight [18.5 −<25], overweight [25 −<30], obese [≥30]), physical activity (inactive [<1h of physical activity/week], medium or high [≥2 h of vigorous and ≥ 2 h of light physical activity/week], low [other]), the prevalence of cardiovascular diseases (yes/no), diabetes (yes/no) and cancer (yes/no). After correction for multiple testing by the false discovery rate (FDR, Benjamini-Hochberg method [48]), CpG sites with corrected *p*-values <0.05 were selected (raw *p*-value <5.4×10^−4^). A Manhattan plot was plotted by the R-package ‘qqman’. Identified sites were then validated in current and never smokers from the validation panel. Loci with replication FDR <0.05 were considered as smoking-associated loci.

To evaluate the impact of cumulative smoking exposure and smoking cessation on DNA methylation, we separately performed additional analyses on the associations of pack-years and time since cessation of smoking with the validated smoking-associated CpG sites in the validation panel. Furthermore, the differences in the methylation of the validated CpG sites were compared for current smokers vs. former smokers and for former smokers vs. never smokers. In all aforementioned analyses, the models were adjusted for covariates as in Model 3 and *p*-values were corrected by FDR (FDR <0.05). Mutual correlations between methylation at the validated CpG sites were assessed by Spearman's correlation coefficients. All data analyses were conducted by SAS version 9.3 (SAS Institute Inc., Cary, NC, USA).

## SUPPLEMENTARY TABLES








