# Supplementary material for: Tobacco smoking and methylation of genes related to lung cancer development
Source: Oncotarget. 2016 Jun 14;7(37):59017–28. doi: 10.18632/oncotarget.10007 (PMC5312292; doi:10.18632/oncotarget.10007)
Supplement: Supplementary file 1 [file oncotarget-07-59017-s001.pdf]

Tobacco smoking and methylation of genes related to lung cancer development

SUPPLEMENTARY TABLES

Supplementary Table S1: Details of selected SNPs and genes (Excel). This table includes four subsets: 1. Lung Cancer Susceptibility Genes Identified by GWASs; 2. Lung cancer SNPs and annotated genes; 3. List of considered CpG sites in/near lung cancer and cis-eQTL genes with exclusion reasons; 4. List of all CpG candidates with mixed linear regression results (Discovery panel).

See Supplementary File 1

Supplementary Table S2: Significant associations between tobacco smoking and methylation of lung cancer related genes in discovery panel.

See Supplementary File 2

Supplementary Table S3: Correlation coefficients between methylation levels at validated CpG sites (Spearman's Rank-Order Correlation) in validation panel.

See Supplementary File 3
