# Supplementary material for: Tobacco smoking and methylation of genes related to lung cancer development
Source: Oncotarget. 2016 Jun 14;7(37):59017–28. doi: 10.18632/oncotarget.10007 (PMC5312292; doi:10.18632/oncotarget.10007)
Supplement: Supplementary file 3 [file oncotarget-07-59017-s003.docx]

**Table S2** **Significant associations between tobacco smoking and methylation of lung cancer related genes in discovery panel ^a^**

| **CpG site** | **Gene** | **Median β value (Q1-Q3)** | | **Effect size ^b^** | **Model 1** | | | **Model 2** | | | **Model 3** | | |
| --- | --- | --- | --- | --- | --- | --- | --- | --- | --- | --- | --- | --- | --- |
|  |  | Never smoker | Current smoker |  | Estimate  (se) | p-value | FDR | Estimate  (se) | *p*-value | FDR | Estimate  (se) | *p*-value | FDR |
| cg00463367 | *GATA3* | 0.235  (0.214-0.257) | 0.226  (0.209-0.242) | -0.009 | -7.6 e-3  (2.9 e-3) | 7.9 e-3 | 0.284 | -9.3 e-3  (2.5 e-3) | 1.8 e-4 | 0.012 | -9.5 e-3 (2.6 e-3) | 3.6 e-4 | 0.044 |
| cg00640087 | *MSH5* | 0.168  (0.145-0.197) | 0.159  (0.135-0.180) | -0.009 | -7.9 e-3  (3.2 e-3) | 0.016 | 0.348 | -9.1 e-3  (1.9 e-3) | 1.7 e-6 | 0.0006 | -7.8 e-3  (2.0 e-3) | 1.4 e-4 | 0.029 |
| cg03281572 | *VTI1A* | 0.839  (0.821-0.857) | 0.827  (0.806-0.844) | -0.012 | -0.0135  (0.003) | 3.7 e-7 | 0.0005 | -0.0123  (0.002) | 4.3 e-7 | 0.0002 | -0.0115  (0.003) | 8.0 e-6 | 0.004 |
| cg03296761 | *MTP18* | 0.631  (0.599-0.657) | 0.626  (0.593-0.668) | -0.005 | -4.0 e-3  (4.2 e-3) | 0.344 | 0.814 | -0.010  (2.6 e-3) | 7.5 e-5 | 0.0064 | -0.011  (2.9 e-3) | 2.0 e-4 | 0.033 |
| cg03420144 | *KCNN3* | 0.771  (0.754-0.788) | 0.769  (0.749-0.790) | -0.002 | -1.1 e-3  (2.6 e-3) | 0.660 | 0.927 | -7.2 e-3  (1.9 e-3) | 1.7 e-4 | 0.012 | -8.3 e-3  (2.1 e-3) | 6.2 e-5 | 0.019 |
| cg04974130 | *KCNIP4* | 0.198  (0.177-0.219) | 0.194  (0.176-0.216) | -0.004 | 4.0 e-3  (3.1 e-3) | 0.202 | 0.713 | 0.010  (2.6 e-3) | 1.7 e-4 | 0.008 | 0.010  (2.8 e-3) | 2.2 e-4 | 0.033 |
| cg06357748 | *RAD52* | 0.632  (0.595-0.671) | 0.651  (0.614-0.685) | 0.019 | 0.019  (4.9 e-3) | 0.0001 | 0.032 | 0.023  (4.8 e-3) | 1.6 e-6 | 0.0006 | 0.020  (5.2 e-3) | 1.2 e-4 | 0.027 |
| cg07269053 | *VTI1A* | 0.746  (0.720-0.775) | 0.723  (0.696-0.756) | -0.023 | -0.0175  (0.004) | 2.6 e-6 | 0.002 | -0.0149  (0.003) | 1.3 e-6 | 0.0005 | -0.0155  (0.003) | 3.2 e-6 | 0.002 |
| cg07745624 | *KCNIP4* | 0.314  (0.279-0.345) | 0.307  (0.283-0.346) | -0.007 | 4.1 e-3  (4.7 e-3) | 0.378 | 0.818 | 0.013  (4.1 e-3) | 1.8 e-3 | 0.054 | 0.017  (4.4 e-3) | 1.2 e-4 | 0.027 |
| cg10163955 | *GATA3* | 0.703  (0.679-0.732) | 0.687  (0.652-0.712) | -0.116 | -0.018  (3.8 e-3) | 1.9 e-6 | 0.0014 | -0.013  (3.3 e-3) | 5.2 e-5 | 0.006 | -0.012  (3.5 e-3) | 3.6 e-4 | 0.044 |
| cg11430077 | *GATA3* | 0.150  (0.129-0.173) | 0.135  (0.119-0.153) | -0.015 | -0.0117  (0.003) | 4.9 e-5 | 0.024 | -0.0129  (0.003) | 4.7 e-6 | 0.001 | -0.0135  (0.003) | 7.6 e-6 | 0.004 |
| cg11743349 | *KCNIP4* | 0.225  (0.199-0.251) | 0.218  (0.196-0.244) | -0.007 | -9.0 e-4  (3.6 e-3) | 0.803 | 0.957 | 8.6 e-3  (2.3 e-3) | 2.3 e-4 | 0.014 | 9.3 e-3  (2.5 e-3) | 2.5 e-4 | 0.036 |
| cg12014547 | *NELL1* | 0.044  (0.038-0.050) | 0.045  (0.039-0.052) | 0.001 | 2.3 e-3  (1.1e-3) | 0.040 | 0.439 | 4.1 e-3  (9.7 e-4) | 2.1 e-5 | 0.003 | 3.6 e-3  (1.0 e-3) | 4.2 e-4 | 0.045 |
| cg12324353 | *TERT* | 0.808  (0.788-0.824) | 0.802  (0.782-0.822) | -0.006 | -2.5 e-3  (2.7 e-3) | 0.345 | 0.814 | -8.0 e-3  (1.9 e-3) | 4.2 e-5 | 0.006 | -7.4 e-3  (2.1 e-3) | 5.1 e-4 | 0.047 |
| cg14602222 | *RAD52* | 0.615  (0.582-0.656) | 0.642  (0.589-0.675) | 0.027 | 0.018  (5.2 e-3) | 0.0005 | 0.074 | 0.023  (5.1 e-3) | 1.1 e-5 | 0.002 | 0.019  (5.5 e-3) | 4.8 e-4 | 0.047 |
| cg16924337 | *RGS17* | 0.063  (0.049-0.082) | 0.065  (0.051-0.083) | 0.002 | 5.8 e-3  (2.9 e-3) | 0.044 | 0.453 | 0.011  (2.4 e-3) | 4.0 e-6 | 0.001 | 9.5 e-3  (2.5 e-3) | 1.9 e-4 | 0.032 |
| cg17928584 | *STK32A* | 0.155  (0.122-0.198) | 0.151  (0.118-0.199) | -0.004 | 1.4 e-3  (5.5 e-3) | 0.806 | 0.959 | 0.014  (3.5 e-3) | 8.0 e-5 | 0.008 | 0.014  (3.8 e-3) | 3.1 e-4 | 0.041 |
| cg18953509 | *MSH5* | 0.098  (0.088-0.110) | 0.091  (0.082-0.101) | -0.007 | -0.0066  (0.001) | 1.7 e-6 | 0.0014 | -0.0064  (0.001) | 1.3 e-7 | 0.0001 | -0.0073  (0.001) | 1.9 e-8 | 5.5 e-5 |

(Continued on following page)

**Table S2** **Significant associations between tobacco smoking and methylation of lung cancer related genes in discovery panel ^a^ (Continued)**

| **CpG site** | **Gene** | **Median β value (Q1-Q3)** | | **Effect size ^b^** | **Model 1** | | | **Model 2** | | | **Model 3** | | |
| --- | --- | --- | --- | --- | --- | --- | --- | --- | --- | --- | --- | --- | --- |
|  |  | Never smoker | Current smoker |  | Estimate  (se) | p-value | FDR | Estimate  (se) | *p*-value | FDR | Estimate  (se) | *p*-value | FDR |
| cg19335412 | *ACTA2* | 0.486  (0.468-0.510) | 0.472  (0.446-0.499) | -0.014 | -0.011  (3.0 e-3) | 1.2 e-4 | 0.032 | -0.014  (2.8 e-3) | 5.8 e-7 | 0.0003 | -0.013  (3.1 e-3) | 4.9 e-5 | 0.017 |
| cg19696491 | *CHRNA5* | 0.493  (0.450-0.526) | 0.504  (0.459-0.544) | 0.011 | 0.012  (5.6 e-3) | 0.032 | 0.433 | 0.018  (4.8 e-3) | 1.3 e-4 | 0.010 | 0.018  (5.1 e-3) | 4.9 e-5 | 0.044 |
| cg19883813 | *GATA3* | 0.094  (0.081-0.107) | 0.086  (0.075-0.098) | -0.008 | -7.3 e-3  (1.9 e-3) | 1.7 e-4 | 0.036 | -7.1 e-3  (1.9 e-3) | 2.6 e-4 | 0.015 | -7.5 e-3  (2.1 e-3) | 3.9 e-4 | 0.044 |
| cg20600210 | *APCDD1* | 0.093  (0.073-0.121) | 0.089  (0.072-0.115) | -0.004 | 2.9 e-3  (3.7 e-3) | 0.426 | 0.834 | 0.012  (2.6 e-3) | 3.5 e-6 | 0.001 | 0.011  (2.8 e-3) | 1.6 e-4 | 0.030 |
| cg20640261 | *MSH5* | 0.454  (0.421-0.486) | 0.430  (0.396-0.467) | -0.014 | -0.0171  (0.004) | 0.0001 | 0.035 | -0.0198  (0.003) | 4.8 e-9 | 1.3 e-5 | -0.0173  (0.004) | 1.5 e-6 | 0.0015 |
| cg22095041 | *CHRNA3* | 0.866  (0.854-0.876) | 0.863  (0.854-0.875) | -0.003 | -2.3 e-3  (1.5 e-3) | 0.131 | 0.642 | -5.0 e-3  (1.4 e-3) | 2.8 e-4 | 0.016 | -5.5 e-3  (1.5 e-3) | 2.0 e-4 | 0.032 |
| cg22563815 | *CHRNA5* | 0.609  (0.577-0.646) | 0.617  (0.585-0.652) | 0.008 | 0.010  (5.0 e-3) | 0.043 | 0.449 | 0.016  (4.3 e-3) | 2.4 e-4 | 0.014 | 0.016  (4.6 e-3) | 4.5 e-4 | 0.045 |
| cg22770911 | *GATA3* | 0.507  (0.485-0.528) | 0.490  (0.465-0.511) | -0.017 | -0.016  (3.1 e-3) | 1.4 e-7 | 0.0004 | -0.012  (2.8 e-3) | 1.7 e-5 | 0.003 | -0.012  (2.9 e-3) | 1.0 e-4 | 0.026 |
| cg24028809 | *RGS17* | 0.298  (0.262-0.331) | 0.275  (0.244-0.316) | -0.023 | -0.0152  (0.004) | 5.8 e-4 | 0.075 | -0.0187  (0.003) | 5.2 e-8 | 7.5 e-5 | -0.0164  (0.004) | 1.5 e-5 | 0.006 |
| cg24258699 | *MSC* | 0.211  (0.187-0.230) | 0.199  (0.176-0.225) | -0.012 | -8.7 e-3  (3.2 e-3) | 6.7 e-3 | 0.281 | -9.6 e-3  (3.1 e-3) | 2.4 e-3 | 0.065 | -0.012  (3.3 e-3) | 4.9 e-4 | 0.047 |
| cg24287110 | *KLF6* | 0.397  (0.354-0.429) | 0.367  (0.336-0.410) | -0.030 | -0.018  (0.005) | 4.7 e-4 | 0.074 | -0.023  (4.3 e-3) | 1.8 e-7 | 0.0001 | -0.022  (4.6 e-3) | 1.4 e-6 | 0.0015 |
| cg24908166 | *TERT* | 0.942  (0.931-0.951) | 0.935  (0.924-0.946) | -0.007 | -5.3 e-3  (1.7 e-3) | 2.0 e-3 | 0.163 | -6.9 e-3  (1.5 e-3) | 4.9 e-6 | 0.001 | -6.6 e-3  (1.6 e-3) | 6.8 e-5 | 0.019 |
| cg26282236 | *RAD52* | 0.616  (0.580-0.654) | 0.636  (0.592-0.668) | 0.020 | 0.016  (5.0 e-3) | 1.2 e-3 | 0.122 | 0.022  (4.9 e-3) | 1.3 e-5 | 0.002 | 0.019  (5.3 e-3) | 2.8 e-4 | 0.039 |

a: Model 1: Adjusted for age (years), sex and random batch effects;

Model 2: Adjusted for covariates in Model 1 plus leukocyte distribution (Houseman algorithm [27]);

Model 3: Adjusted for covariates in Model 2, plus alcohol consumption (abstainer/ low/ intermediate/ high), body mass index (BMI, underweight/ normal weight/ overweight/ obese), physical activity (inactive/ low/ medium or high), prevalence of cardiovascular diseases (yes/no), prevalence of diabetes (yes/no) and prevalence of cancer (yes/no);

All 2854 probes were tested by the three models, and the threshold of FDR is 0.05. A total of 31 CpG sites were identified to be associated with smoking exposure;

b: Effect size = Median β_current smoker_ – Median β_never smoker_
