# Supplementary material for: Tobacco smoking and methylation of genes related to lung cancer development
Source: Oncotarget. 2016 Jun 14;7(37):59017–28. doi: 10.18632/oncotarget.10007 (PMC5312292; doi:10.18632/oncotarget.10007)
Supplement: Supplementary file 4 [file oncotarget-07-59017-s004.docx]

**Table S3 Correlation coefficients between methylation levels at validated CpG sites (Spearman's Rank-Order Correlation) in validation panel ^a^**

| **CpG site** |  | **CpG site** | | | | | | | | | | | | |
| --- | --- | --- | --- | --- | --- | --- | --- | --- | --- | --- | --- | --- | --- | --- |
|  |  | **cg17928584** | **cg12324353** | **cg24908166** | **cg00640087** | **cg20640261** | **cg19335412** | **cg10163955** | **cg11430077** | **cg22770911** | **cg24287110** | **cg03281572** | **cg07269053** | **cg19696491** |
|  | **Gene** | *STK32A* | *TERT* | *TERT* | *MSH5* | *MSH5* | *ACTA2* | *GATA3* | *GATA3* | *GATA3* | *KLF6* | *VTI1A* | *VTI1A* | *CHRNA5* |
| **cg17928584** | *STK32A* | 1.000 | -0.540^***^ | -0.354^***^ | 0.118^**^ | 0.018 | -0.242^***^ | 0.110^*^ | 0.005 | 0.220^***^ | -0.301^***^ | 0.145^**^ | 0.249^***^ | 0.317^***^ |
| **cg12324353** | *TERT* | -0.540^***^ | 1.000 | 0.502^***^ | -0.167^**^ | -0.016 | 0.281^***^ | 0.110^*^ | 0.125^**^ | 0.014 | 0.358^***^ | -0.028 | -0.075 | -0.256^***^ |
| **cg24908166** | *TERT* | -0.354^***^ | 0.502^***^ | 1.000 | -0.284^***^ | -0.136^**^ | 0.050 | 0.189^***^ | 0.013 | 0.079 | 0.125^**^ | -0.088^*^ | -0.142^**^ | -0.249^***^ |
| **cg00640087** | *MSH5* | 0.118^**^ | -0.167^**^ | -0.284^***^ | 1.000 | 0.707^***^ | 0.412^***^ | -0.274^***^ | 0.124^**^ | -0.057 | -0.029 | 0.209^***^ | 0.333^***^ | 0.374^***^ |
| **cg20640261** | *MSH5* | 0.018 | -0.016 | -0.136^**^ | 0.707^***^ | 1.000 | 0.467^***^ | -0.047 | 0.137^**^ | 0.130^**^ | 0.079 | 0.324^***^ | 0.397^***^ | 0.453^***^ |
| **cg19335412** | *ACTA2* | -0.242^***^ | 0.281^***^ | 0.050 | 0.412^***^ | 0.467^***^ | 1.000 | 0.087^*^ | 0.291^***^ | 0.202^***^ | 0.302^***^ | 0.181^***^ | 0.212^***^ | 0.201^***^ |
| **cg10163955** | *GATA3* | 0.110^*^ | 0.110^*^ | 0.189^***^ | -0.274^***^ | -0.047 | 0.087^*^ | 1.000 | 0.376^***^ | 0.795^***^ | 0.192^***^ | 0.321^***^ | 0.165^***^ | 0.207^***^ |
| **cg11430077** | *GATA3* | 0.005 | 0.125^**^ | 0.013 | 0.124^**^ | 0.137^**^ | 0.291^***^ | 0.376^***^ | 1.000 | 0.469^***^ | 0.364^***^ | -0.009 | 0.018 | 0.063 |
| **cg22770911** | *GATA3* | 0.220^***^ | 0.014 | 0.079 | -0.057 | 0.130^**^ | 0.202^***^ | 0.795^***^ | 0.469^***^ | 1.000 | 0.257^***^ | 0.285^***^ | 0.214^***^ | 0.325^***^ |
| **cg24287110** | *KLF6* | -0.301^***^ | 0.358^***^ | 0.125^**^ | -0.029 | 0.079 | 0.302^***^ | 0.192^***^ | 0.364^***^ | 0.257^***^ | 1.000 | -0.071 | -0.137^**^ | -0.075 |
| **cg03281572** | *VTI1A* | 0.145^**^ | -0.028 | -0.088^*^ | 0.209^***^ | 0.324^***^ | 0.181^***^ | 0.321^***^ | -0.009 | 0.285^***^ | -0.071 | 1.000 | 0.549^***^ | 0.364^***^ |
| **cg07269053** | *VTI1A* | 0.249^***^ | -0.075 | -0.142^**^ | 0.333^***^ | 0.397^***^ | 0.212^***^ | 0.165^**^ | 0.018 | 0.214^***^ | -0.137^**^ | 0.549^***^ | 1.000 | 0.441^***^ |
| **cg19696491** | *CHRNA5* | 0.317^***^ | -0.256^***^ | -0.249^***^ | 0.374^***^ | 0.453^***^ | 0.201^***^ | 0.207^***^ | 0.063 | 0.325^***^ | -0.075 | 0.364^***^ | 0.441^***^ | 1.000 |

a: Categories of *p*-values for the correlation coefficients: *** : *p*<0.0001 ; **: *p* <0.01 ; * : *p* <0.05 ; none: *p* ≥0.05
